# What factors influence level-2 visual perspective-taking ability in 4-year-old children?

**DOI:** 10.3389/fpsyg.2026.1867009

**Published:** 2026-10-28

**Authors:** Jinxia Ding, Jiahui Chen, Jiahui Bao, Jieyu Lin, Lulu Jia, Penghui Hu, Xiaotong Liu, Junhua Jiang, Junfeng Diao

**Affiliations:** 1 School of Education, Hainan Normal University, Haikou, China; 2 Key Laboratory of Data Science and Smart Education of Ministry of Education, Hainan Normal University, Haikou, China; 3 School of Foreign Languages, Hainan Normal University, Haikou, China

**Keywords:** level-2 visual perspective taking, 4 -year-olds, picture-taking task, rotation angle, test tool, egocentric errors

## Abstract

**Introduction:**

Level-2 visual perspective taking (VPT-2) reflects children’s ability to understand others’ visual experiences. However, the effects of viewing angle and task materials on 4-year-olds’ performance remain unclear.

**Methods:**

Using a picture-taking (“photographer”) task, this study examined the effects of test tool and rotation angle, as well as egocentric error patterns, among 163 four-year-old children from Hainan, China. A 2 (test tool: Lego figurines vs. geometric solids) × 3 (rotation angle: 0°, 90°, 180°) within-subjects design was used. The formal test comprised 18 four-alternative trials, with three trials in each Tool × Angle condition; each correct response scored 1 and each incorrect response scored 0. Performance was assessed using raw correct scores and the proportions of egocentric errors.

**Results:**

Rotation angle significantly affected performance in a nonmonotonic pattern: raw correct scores were highest at 0°, followed by 180°, and lowest at 90°. The main effect of test tool was significant, with higher raw correct scores in the Lego figurine condition than in the geometric solids condition. The Tool × Angle interaction for raw correct scores was not significant. The proportions of egocentric errors showed significant main effects of test tool and rotation angle, as well as a significant Tool × Angle interaction. Egocentric errors were most pronounced in the geometric solids condition at 180°. Supplementary analyses revealed no significant sex main effect or sex-related interactions.

**Discussion:**

These findings indicate that 4-year-olds’ VPT-2 performance varies across rotation angles and test materials. Performance differences between material conditions may be associated with differences in task structure, stimulus discriminability, social cues, and spatial-relational judgment demands, whose independent contributions could not be isolated in this study. Because the analyses relied on raw correct scores and error-type data, the findings should be interpreted as differences in behavioral performance rather than direct evidence about processing strategies or cognitive mechanisms.

## Introduction

### Conceptualizing visual perspective taking and related research

Visual perspective taking (VPT) is a social-cognitive ability that allows children to understand that others’ perceptual experiences may differ from their own; its roots trace back to the “three mountains” task of Piaget and Inhelder (1956). In that task, young children, when asked to judge what another person sees of a spatial layout, were prone to responses influenced by their own viewpoint—that is, a degree of egocentrism. Although the three mountains task focused on spatial perspective understanding, the core question it posed—whether children can go beyond their own viewpoint to understand another person’s visual experience—laid the theoretical foundation for subsequent research on VPT.

As the field developed, research on VPT shifted from general spatial perspective understanding to specific examinations of others’ visual experiences. VPT is commonly defined as the ability to mentally construct another person’s viewpoint, distinct from one’s own, and on that basis to infer what that person can see and how an object appears to them (Ford et al., 2023; Frick et al., 2014; Michelon and Zacks, 2006). VPT is thus not merely a judgment about the positions of objects; it involves children’s understanding of the cognitive state that others may have visual experiences different from their own.

Building on children’s performance across different visual inference tasks, Flavell et al. (1981) further proposed a developmental distinction between level-1 visual perspective taking (VPT-1) and level-2 visual perspective taking (VPT-2).

VPT-1 primarily involves judging another person’s visible field—that is, understanding whether an object falls within another person’s line of sight. For example, when an observer changes position, children must judge whether the observer can see a given object. Such tasks mainly require children to understand *whether* others can see something.

VPT-2, by contrast, further requires children to understand that even when two individuals look at the same object, their visual experiences may differ because of their different viewing positions. Children must therefore judge not only whether another person can see something but also infer the specific appearance or spatial relations the object presents from that person’s viewpoint. Because this process involves inhibiting one’s own perspective, representing another person’s perspective, and comparing different visual experiences, its task demands are typically more complex (Kessler and Rutherford, 2010). Michelon and Zacks (2006) further distinguished visibility judgments from judgments of relative spatial orientation, suggesting that the two types of tasks may engage different cognitive processes. Research in China has likewise focused on the distinction between self and other perspectives. Wu W. et al. (2022) emphasized the mental representation of others’ viewpoints in VPT and found, using a negative-priming experiment, that when self and other perspectives were incongruent, the perspective-taking task involved inhibiting one’s own perspective. Wu M. et al. (2022) discussed the processing mechanisms of perspective conflict from the standpoint of egocentric bias. Together, these studies indicate, from the perspectives of developmental performance and cognitive processing alike, that understanding others’ visual experiences involves not only identifying another person’s viewing position but also, potentially, coordinating self and other perspectives. Behavioral findings by Moll et al. (2013) provide further evidence for this distinction in task demands. That study compared the performance of 3- and 4.5-year-olds on two tasks involving color filters. In a perspective-taking task that required only selecting targets according to an adult’s visual perspective, 3-year-olds answered correctly on 71% of trials, significantly above chance; in a perspective-confronting task that required explicitly judging and comparing their own and the adult’s differing visual experiences, however, their accuracy was only 44%, not significantly above chance. By comparison, the 4.5-year-olds achieved 79 and 74% on the two tasks, respectively. This dissociation indicates that being able to select responses according to another person’s visual perspective does not necessarily mean that children can simultaneously represent and compare conflicting visual experiences of self and other. The difficulty of VPT-2 tasks may thus stem not only from establishing a representation of another person’s viewpoint but also from the requirement to coordinate self and other perspectives. These behavioral differences, however, support a distinction in task demands and do not warrant direct inference about mutually independent cognitive mechanisms.

From the perspective of social-cognitive development, VPT is closely related to theory of mind (ToM). ToM concerns the understanding of others’ beliefs, desires, knowledge, and other mental states, whereas VPT concerns others’ perceptual experiences; both involve recognizing differences between one’s own and others’ experiences. In his review of children’s ToM development, Flavell (1999) incorporated children’s understanding of visual perception into research on the development of mental-world knowledge. Using a colored-filter task, Moll and Meltzoff (2011) further found that under certain conditions 3-year-olds could already judge how an object appears from another person’s viewpoint, and they discussed the links between this ability, the appearance–reality distinction, and false-belief understanding. Level-2 visual perspective taking is thus theoretically related to other ToM abilities, although the specific cognitive demands of different tasks are not identical; VPT-2 performance should therefore not be equated with children’s overall level of ToM development.

Regarding the relation between visual and spatial perspective taking, some studies in China have adopted broader conceptualizations that subsume judgments of spatial features or spatial relations induced by another person’s viewing position under the rubric of perspective taking (Wu and Cao, 2019; Zhang and Wang, 2008). This line of research is related to the VPT tradition that emphasizes others’ visual experiences, but the two research domains are not fully overlapping. Specifically, VPT concerns what an observer can see and how objects appear from the observer’s viewpoint, whereas spatial perspective taking may additionally involve broader judgments of spatial orientation and position relations. Building on Flavell et al.’s (1981) distinction between VPT-1 and VPT-2 and on the picture-taking paradigm of Frick et al. (2014), the present study adopts the theoretical framework of VPT-2, focusing on whether children can judge the visual appearance or spatial relations of objects according to another person’s viewing position.

In sum, although the studies reviewed above differ in theoretical focus and task operations, all concern how individuals understand their own and others’ viewing experiences. Piaget and Inhelder (1956) focused on children’s egocentric tendency when judging others’ spatial viewpoints; Flavell et al. (1981) distinguished VPT-1 from VPT-2 on the basis of the difference between visibility judgments and visual-appearance judgments; subsequent research has further examined how perspective transformation, spatial frames of reference, and specific task demands relate to perspective-taking performance. These studies provide evidence on VPT at different levels, but they do not imply that all spatial viewpoint judgment tasks involve the same cognitive processes. The present study therefore adopts the VPT-2 framework, uses a picture-taking task to examine children’s judgments of visual appearance and spatial relations based on another person’s viewing position, and limits its conclusions to behavioral differences under specific task conditions.

### Performance of preschool children on visual perspective-taking tasks and research paradigms

Existing evidence indicates that preschool children have begun to show the ability to understand others’ visual experiences, but different types of VPT tasks may differ in difficulty. Flavell et al. (1981) examined VPT in 51 3-year-olds across three experiments. In the first, 3-year-olds were given level-1 and level-2 tasks. In the level-1 tasks, children judged whether the experimenter seated opposite them could see the dog or the cat on a two-sided card, or which parts of an occluded turtle picture were visible to the experimenter; in the level-2 task, children judged whether the same turtle picture appeared upright or inverted from the experimenter’s viewpoint. Children answered the level-1 questions correctly, but their accuracy dropped markedly in the level-2 task. This difference indicates that being able to judge what others see does not imply being able to judge accurately how objects appear from others’ viewpoints, providing empirical support for the distinction in task demands between the two levels of visual perspective taking.

Using a colored-filter task, Moll et al. (2013) further examined preschool children’s VPT; the pilot included 27 3-year-olds, and the main study included 112 children aged 3 and 4.5 years. Children’s performance changed depending on whether the task required explicitly comparing their own and others’ visual experiences, further indicating that preschool children’s difficulty on VPT-2 tasks is markedly dependent on task demands.

The verbal demands of traditional VPT tasks may also affect young children’s performance. Some tasks require children not only to understand object appearance or spatial orientation but also to simultaneously process the relations among the observing agent, the viewing position, and the response options. For example, in the colored-filter task, Moll et al. (2013) asked children, “How do I see the dog from over here—do I see it blue or do I see it green?,” with a corresponding question about the color the children themselves saw. Such questions require children to identify person references such as “I/you,” to distinguish different viewing positions, and to map each perspective onto a color judgment. For children whose language comprehension is still developing, these additional verbal-processing demands may affect performance, so that the results reflect both perspective-taking ability and task comprehension. It is therefore advisable in research design to minimize, as far as possible, verbal load unrelated to the core perspective judgment. The picture-taking paradigm of Frick et al. translates VPT-2 into a picture-selection task via the three-term relation of photographer–object–photograph: children respond mainly by pointing to a candidate picture and need not describe their reasoning verbally. This paradigm thus reduces verbal output demands while preserving the core requirement of judging an object’s visual appearance from another person’s viewing position. Moreover, Frick et al. tested 80 children aged 4–8 years and found that VPT-2 performance improved with age, with 4-year-olds still showing substantial difficulty under conditions requiring perspective transformation—indicating that the paradigm is well suited to examining the developmental characteristics of VPT-2 in the preschool period.

### Rotation angle, task materials, and the present study

Rotation angle is an important variable in VPT tasks. In the 0° condition, the child’s and the observer’s viewpoints coincide, and no marked perspective transformation is typically required; in the 90° and 180° conditions, children must re-judge the object’s visual presentation according to the other person’s viewing position. In the picture-taking study of Frick et al. (2014), children’s performance was affected by viewpoint disparity and scene complexity. Related work by Surtees et al. (2013) and Kessler and Rutherford (2010) provides further theoretical reference for understanding the perspective-transformation demands of different perspective-taking tasks. Using a model-city navigation task with 245 children aged 8–12 years, Vander Heyden et al. (2017) found that accuracy was lower and response times longer at 90° than at 180°. This suggests that task difficulty does not necessarily increase linearly with angular disparity; that study, however, involved older children and a task format different from the present one, so the same performance pattern cannot be assumed for 4-year-olds. The present study therefore included 0°, 90°, and 180° conditions to test whether 4-year-olds show systematic performance differences across viewpoint relations.

The characteristics of task materials may also influence children’s VPT-2 performance, and such influence is not limited to whether the materials carry social features; it may also involve the explicitness of visual cues and the spatial-relational judgments the task requires. In two experiments on adult spatial perspective taking with 120 and 185 participants, respectively, Gunalp et al. (2019) compared the effects of human-like avatars, arrows, and chairs as cues for spatial viewpoint judgment. Performance was better with human-like avatars than with abstract arrows and control conditions, whereas no significant difference emerged between the chair and avatar conditions, both of which outperformed the control condition. This indicates that merely providing directional cues does not necessarily improve perspective-taking performance; whether observers can use familiar objects and their spatial orientation to establish an imagined viewing position may also affect task completion. That study, however, used an adult spatial-orientation task, so its results provide only indirect evidence for understanding the potential roles of different stimulus features and cannot directly support inferences about 4-year-olds’ cognitive mechanisms. In children’s VPT tasks, material characteristics may additionally alter the visual information that must be processed for the judgment.

The research of Frick et al. (2014) further indicates that children’s picture-taking performance is affected by viewing angle and scene complexity. The Lego figurines used in the present study have clear facial, body-orientation, and posture features, allowing children to distinguish the object’s appearance across viewpoints on the basis of these visible features; by contrast, the geometric condition requires children to judge the relative spatial relations of two objects according to the photographer’s position, combining the positions of multiple objects and their interrelations when making a selection. In addition, some geometric solids are rotationally symmetric, but correct judgments in the geometric condition rest mainly on the relative spatial relations between objects; task difficulty there may thus be affected not only by the discriminability of a single object’s appearance but also by object combinations and the demands of spatial-relational judgment. The two types of materials may therefore produce performance differences because of differences in visual cues, number of objects, and judgment demands, but the independent contributions of these factors remain to be tested.

On this basis, the present study adopted the picture-taking paradigm of Frick et al. (2014) to examine 4-year-olds’ VPT-2 performance across rotation angles and task materials. Four-year-olds were chosen as participants primarily because of the developmental characteristics they show on perspective-taking tasks. Frick et al. (2014) tested 80 children aged 4–8 years; the 16 four-year-olds averaged 35% correct, whereas the 8-year-olds averaged 76%. These results indicate that 4-year-olds can participate in experimental procedures based primarily on picture selection, yet still face marked difficulty on tasks requiring judgments of object appearance from another person’s position. Meanwhile, the colored-filter research of Moll et al. (2013) showed that 3-year-olds could already make judgments according to another person’s visual perspective when the task did not require directly comparing their own and others’ perspectives, but their performance remained clearly limited when the task required simultaneously representing and comparing their own and others’ differing visual experiences. Together, these findings indicate that 4-year-olds can participate in picture-selection perspective-taking tasks, although their performance remains contingent on specific task demands. The present study therefore focused on 4-year-olds to examine performance differences across rotation angles and task materials. Practice trials with feedback were included to help children become familiar with the procedure and to reduce errors arising from misunderstanding the basic rules. A 2 (test tool: Lego figurines vs. geometric solids) × 3 (rotation angle: 0°, 90°, 180°) within-subjects design was used, with 0° as a no-transformation baseline condition and 90° and 180° as conditions with incongruent viewpoints. It should be noted that, although both conditions used the picture-taking paradigm, they involve somewhat different stimulus features and judgment demands. The Lego condition mainly requires children to judge the appearance and orientation of a single human-like object across viewpoints, whereas the geometric condition requires them to judge the relative spatial relations between two objects. The two conditions differ not only in the social features of the photographed objects but also, simultaneously, in number of objects, visual discriminability, and spatial-relational judgment demands. Because these factors were neither separately manipulated nor independently controlled in the present design, even significant score differences between the two conditions cannot be attributed specifically to the human-like features, stimulus discriminability, or the differing demands of spatial-relational judgment. The material conditions were therefore treated as holistic experimental conditions bundling multiple task characteristics, and the study primarily compared children’s behavioral performance across the two conditions rather than making causal inferences about the independent role of any specific factor.

In sum, prior research indicates that VPT-2 is an important index of preschool children’s perspective-taking development and suggests that rotation angle, task material, and spatial-relational demands may affect children’s performance. Three gaps remain, however. First, angle-specific evidence for 4-year-olds is still insufficient; in particular, whether 90° and 180° show a nonlinear difference requires testing. Second, whether VPT-2 performance differs across material conditions, and whether such differences indicate task-context dependence, requires further examination. Third, egocentric errors are often treated generically as a manifestation of VPT-2 failure, but whether they vary across tool and angle conditions has lacked fine-grained analysis. Addressing these gaps, the present study examined 4-year-olds’ behavioral performance across rotation angles and test materials using a picture-taking task, and further analyzed condition differences in egocentric errors, thereby supplementing the empirical evidence on preschool children’s VPT-2 task performance.

### Research questions

Building on the foregoing research, this study examined 4-year-olds’ performance differences on VPT-2 tasks with respect to rotation angle, test material, and egocentric errors.

*Research Question 1*: Does rotation angle affect 4-year-olds’ VPT-2 task performance?

Prior work suggests that VPT task performance may vary with viewing angle (Frick et al., 2014). Vander Heyden et al. (2017) further found that children’s performance at 90° and 180° may differ nonlinearly, but that result comes from older children and is not sufficient to directly predict the performance pattern of 4-year-olds.

*H1*: Four-year-olds’ VPT-2 task performance differs across rotation angles, with correct scores at 0° expected to be higher than at 90° and 180°. The difference between 90° and 180° was examined as an exploratory question.

*Research Question 2*: Do different test materials affect 4-year-olds’ VPT-2 task performance?

The research of Frick et al. (2014) indicates that scene complexity is related to children’s VPT performance. Given that the present Lego figurine condition and geometric solids condition differ in visual cues, number of objects, and spatial-relational judgment demands, the two tasks may differ in difficulty.

*H2*: Four-year-olds are expected to obtain higher correct scores in the Lego figurine condition than in the geometric solids condition. This prediction concerns overall performance across the two task conditions and does not involve the independent effect of any specific material feature.

*Research Question 3*: Do egocentric errors vary with test material and rotation angle?

This study further examined whether, under incongruent-viewpoint conditions, egocentric errors differ across test materials and rotation angles. Because existing evidence is not yet sufficient to support a clear directional prediction, this analysis was exploratory.

In addition, sex was included only as a supplementary variable, to check whether the main tool and angle effects were influenced by sex. Because sex differences were not the core research question, and because conventional null-hypothesis significance testing cannot treat “no sex difference” as a research hypothesis, no sex-related hypothesis was advanced. If neither a significant sex main effect nor significant sex-related interactions emerged, no sex-related *post hoc* comparisons would be conducted.

## Method

### Participants

Data collection for this study was initially conducted under the “1 + *N*” program of the pilot zone for improving childcare and education quality in kindergartens of Hainan Province, China, which provided the kindergarten contacts, participant recruitment, and on-site testing conditions. The subsequent data management and analysis related to this study were conducted under the Hainan Provincial Educational Science Planning commissioned project “Curriculum Model Construction and Practice Paths for High-Quality Kindergarten Development in Hainan Province” (Grant No. QJW20250404). Using stratified random sampling, middle-class children (the kindergarten class for 4- to 5-year-olds) were randomly recruited from public kindergartens in urban and township areas of four cities and counties of Hainan Province—Haikou, Danzhou, Wanning, and Qiongzhong Li and Miao Autonomous County—after their legal guardians signed written informed consent forms.

Inclusion criteria were as follows: (a) aged at least 4.0 years and younger than 5.0 years; (b) no history of neurodevelopmental disorders or visual impairment; (c) able to understand the Mandarin test instructions and respond accordingly; and (d) written informed consent signed by a legal guardian.

A total of 190 children were tested, of whom 27 were excluded because they did not meet the age criterion; the remaining 163 all completed testing, with no dropout or noncompliance. The final effective sample comprised 163 children (80 boys, 83 girls) aged 48–59 months (*M* = 4 years 4 months, *SD* = 3.2 months).

An *a priori* power analysis was conducted using G*Power 3.1. For a 2 × 3 repeated-measures ANOVA (within-subjects design) with a medium effect size (*f* = 0.25), α = 0.05, power (1 − β) = 0.80, 6 measurements, and an estimated inter-measure correlation of 0.3, the required minimum sample size under the sphericity assumption was 128.

### Design

A 2 (test tool: Lego blocks vs. geometric solids) × 3 (rotation angle: 0° vs. 90° vs. 180°) fully repeated-measures design was adopted, with both factors as within-subject variables. The 0° condition served as a no-transformation baseline, used to check children’s basic understanding of the task rules. Throughout the experiment, the child remained in a fixed position (facing the front of the display); the angular disparity between perspectives was manipulated by changing the spatial position of the “photographer” figurine.

#### Independent variables

- Rotation angle: the angular disparity between the participant’s and the observer’s (photographer’s) viewpoints was 0° (same direction), 90° (orthogonal), or 180° (opposite), as illustrated in Figures 1–3, respectively.
- Test tool: Lego blocks (human-like figurines) versus geometric solids (abstract geometric objects).

**Figure 1:**
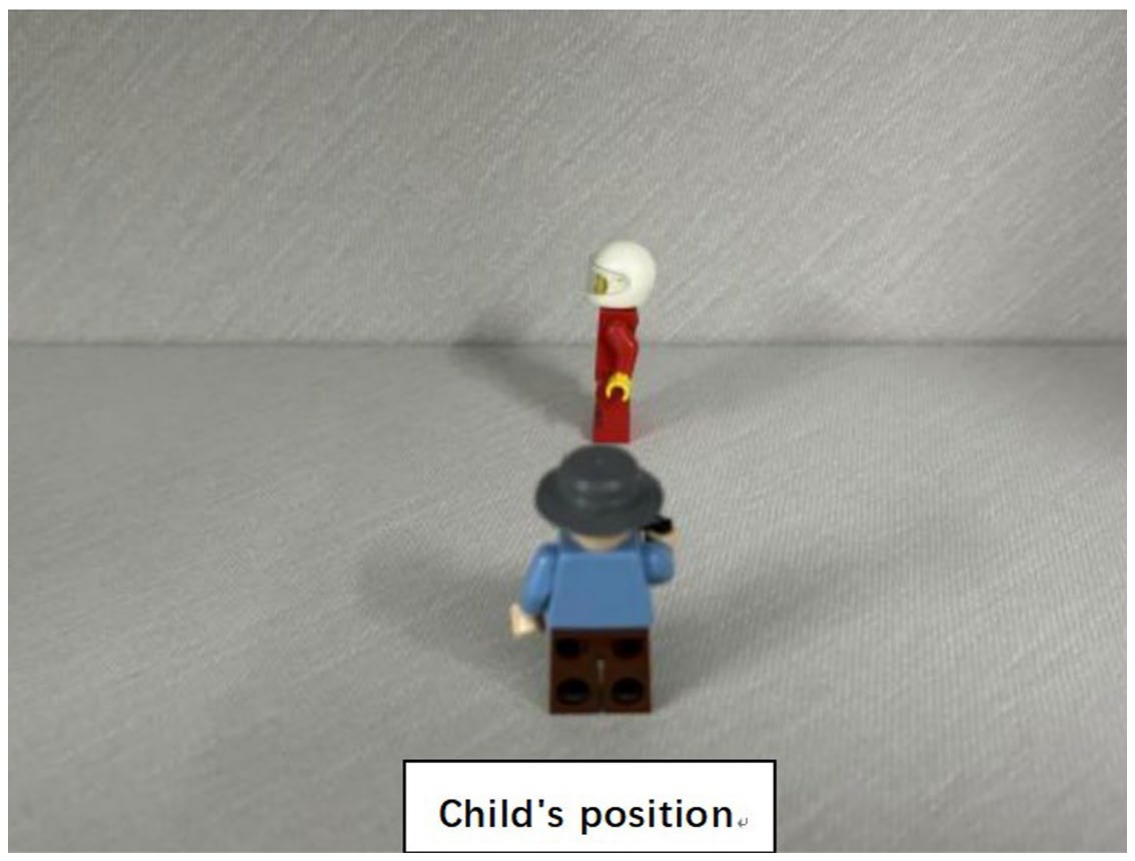
The observation object and the subject are at 0°.

**Figure 2:**
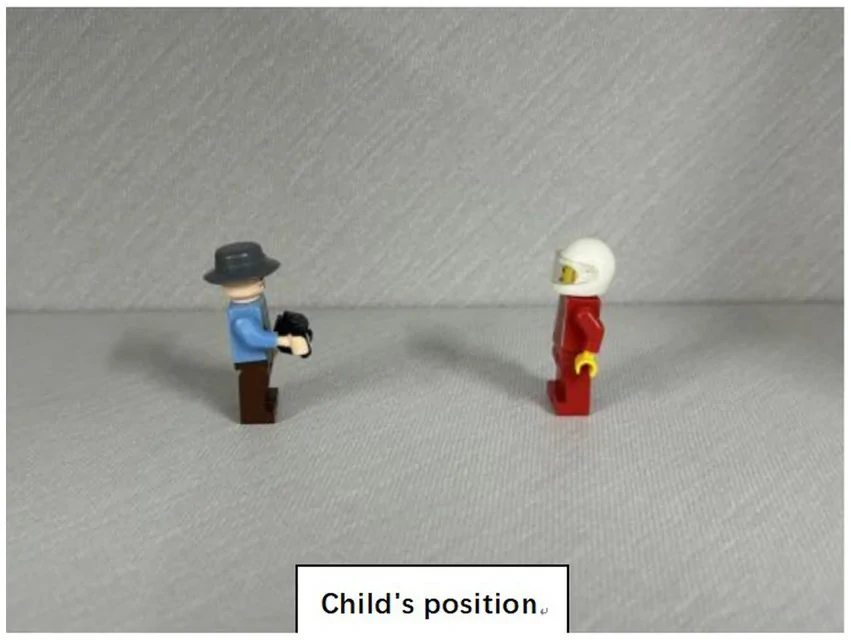
The observation object and the subject are at 90°.

**Figure 3:**
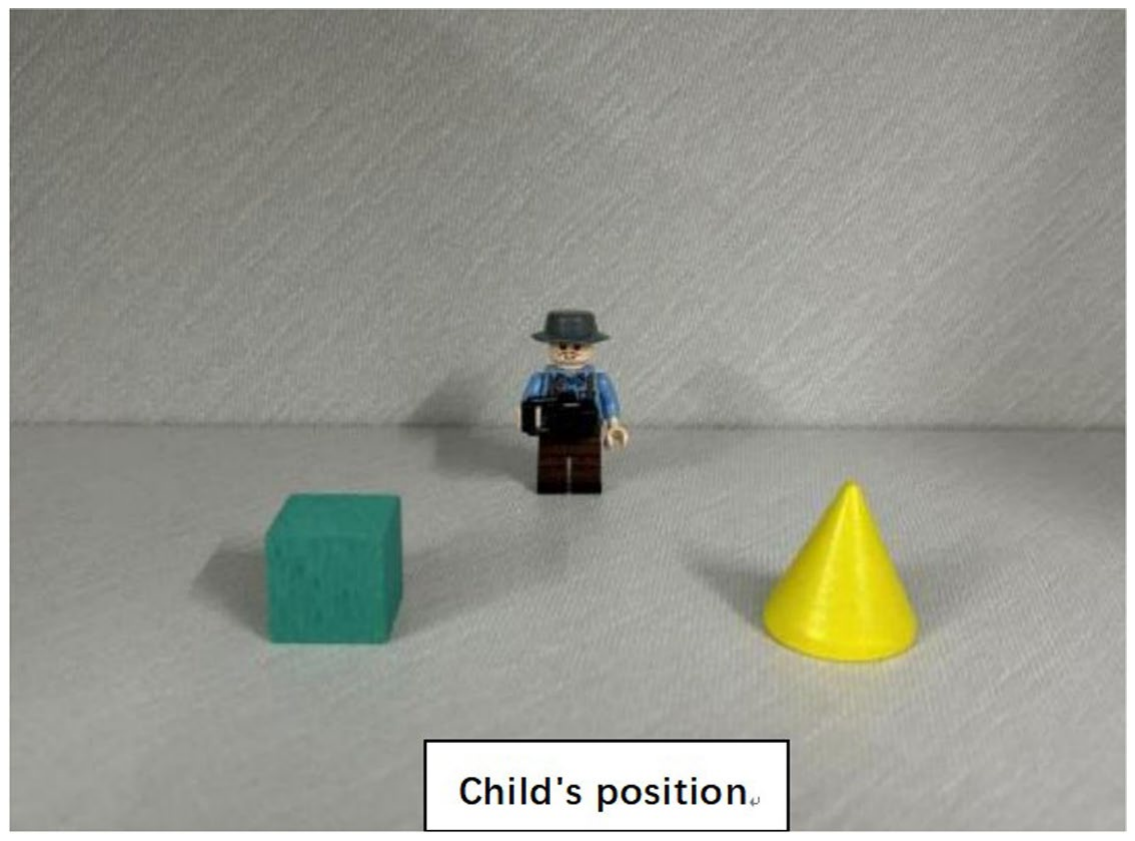
The observation object and the subject are at 180°.

The dependent variable was the number of correct responses (raw correct score) in each Tool × Angle condition. Each child received six condition scores—Lego 0°, Lego 90°, Lego 180°, geometric 0°, geometric 90°, and geometric 180°. Because the Lego and geometric tasks differ in task structure, stimulus discriminability, and social cues, and because the two tool scores were only weakly correlated, the two tools were treated as related but nonequivalent task conditions and analyzed separately, rather than being combined into a single VPT-2 total score.

#### Scoring and response coding procedure

The formal test comprised 18 trials in a 2 (test tool: Lego blocks, geometric solids) × 3 (rotation angle: 0°, 90°, 180°) × 3 (repeated trials) arrangement, with 3 trials in each Tool × Angle condition. Each trial was a four-alternative picture-selection task, and the correct option for each trial was determined in advance, before testing, according to the photographer’s position and the corresponding viewing angle. Formal scoring thus involved no subjective judgment by experimenters of children’s perspective-taking performance.

After viewing the four candidate photographs, children made their choice by indicating one photograph, and the experimenter recorded the final selection. If a child’s first indication did not clearly correspond to a single photograph, the experimenter merely asked the child to point again clearly to one photograph, providing no information or hints about the correct answer; the single photograph the child ultimately and clearly indicated served as the formal response for that trial. No feedback on correctness was provided during the formal test phase.

After data entry, children’s selections were checked trial by trial against the predetermined answer key. A selection consistent with the standard answer for that trial scored 1, and an inconsistent selection scored 0. Scoring was thus a direct match between the child’s actual choice and the preset answer and involved no subjective rating of children’s verbal expression, gestures, or response quality. The raw correct score for each Tool × Angle condition was the sum of correct responses across the 3 trials, ranging from 0 to 3; the total across the 9 formal trials of each tool ranged from 0 to 9. The theoretical range of the whole-task total was 0 to 18, but given the differences between the two tools in task structure and stimulus features, the whole-task total was not used as a primary analytic index.

All 163 children included in the final analyses completed all 18 formal trials, with no missing trials or uncodable formal responses; no missing-value adjustment or weighting by number of completed trials was therefore required. Practice trials served only to familiarize children with the task rules; feedback could be given during practice, but practice responses were not counted toward formal scores.

#### Control variables

- Object presentation side (left–right counterbalancing).
- Position of the candidate photographs (randomized).
- Standardization of instructions (uniform training).

### Experimental materials

### Lego block materials

Standard Lego figurines (approximately 4 cm tall) were used, with the face oriented forward and the limbs extended symmetrically, to maximize the identifiability of the face and orientation cues.

### Geometric solid materials

The geometric condition used a set of solid blocks, comprising yellow cones, yellow cylinders, green/pink/yellow/blue cubes, and wood-colored rectangular prisms. On each trial, two objects were selected from the set and placed on the table, forming a three-element scene with the photographer figurine (see Figure 3).

In the geometric condition, the task required participants to judge the spatial relation between the two objects (e.g., left–right or front–back relations) from the photographer’s viewpoint, rather than to judge changes in the visual orientation of a single object. Scoring was therefore based on the consistency of the relative spatial positions of the objects, not on the appearance change of a single stimulus across viewpoints. Rotationally symmetric objects such as cones and cylinders thus did not affect the definition of correct answers, because rotation across different angles does not alter the judgment of relative spatial relations between objects.

### Candidate photographs

The experimental materials were photographed from the viewpoints the photographer might occupy—front, left side, right side, and back—yielding the four candidate photographs used in each trial. Before presentation, the photographs were uniformly cropped and laid out to remove, as far as possible, background information irrelevant to the judgment, while retaining the objects themselves and the appearance or spatial-relational information needed for the viewpoint judgment. The four candidate photographs were presented simultaneously in a 2 × 2 array on a landscape A4 sheet, each photograph approximately 11 cm × 8 cm, clearly separated and numbered so that children could recognize and indicate them. The positions of the four candidates were randomized across trials to reduce the influence of fixed-position preference on selection (see Figure 4).

**Figure 4:**
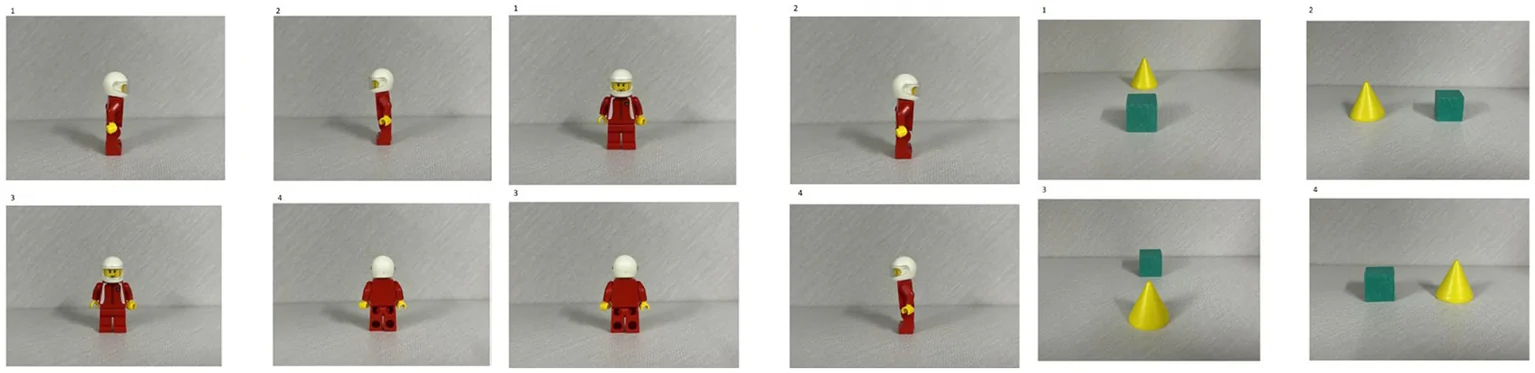
Collection of photos for the participants to choose from.

To ensure that children understood what the photographs depicted and how they corresponded to the actual scene, three practice trials preceded the formal test. The practice used the same four-alternative picture-selection format; the experimenter explained the photographer’s position and the relations among the actual objects and the candidate photographs, and asked children to point to the photograph the photographer would “take” from the current position. If a child chose incorrectly during practice, the experimenter gave corrective feedback and restated the task requirements; practice trials were not scored. Once formal testing began, no feedback on correctness was provided.

### Procedure

#### Experimenter training and double-blind control

All experimenters (*n* = 10) received 3 h of standardized training before the experiment, covering (a) memorized administration of the instructions, (b) skills for interacting with young children, and (c) protocols for handling unexpected situations. A double-blind design was used: experimenters were unaware of participants’ specific background information (sex, exact age), and participant assignment information was managed by an independent coder.

#### Within-trial randomization

Within each trial, the positions of the four candidate photographs were determined by a random number table, to avoid position-preference effects.

#### Testing sequence

The experiment was conducted individually in a quiet, distraction-free room. Before the formal test, the experimenter first exchanged a few words with the child—asking the child’s name and class, among other basics—to gain an initial sense of the child’s comprehension of and responses to everyday questions in Mandarin. The sequence then proceeded as follows:

- The experimenter gave the child a brief introduction to the task: “Let us play a picture-taking game. Let us try it first: the photographer stands here, and the child/blocks are here. The photographer takes a picture of the child/blocks from this angle—which picture comes out? Please point to it for me.”
- Photographs were presented in random order after each “shot” was completed, with no fixed order; after viewing them, the child pointed to the picture that had been “taken,” and the experimenter recorded the child’s choice. (The first three “shots,” which used a boy figurine, were practice trials; during practice the experimenter gave feedback, and when the child chose incorrectly the experimenter pointed out the error and guided the child to the correct photograph. Practice responses were not scored.)
- The experimenter recorded the child’s response on each trial; each correct response earned 1 point and each incorrect response earned none, for 18 trials in total.
- After the session, the child was thanked and given a small gift as a reward.

The entire experiment took approximately 10 min per child. Because the session was short, no fixed breaks were scheduled during formal testing. Interruptions were rare in practice; if a child needed to leave temporarily—to drink water, use the toilet, or for other personal reasons—the experimenter paused the test and resumed from the point of interruption once the child was ready, without requiring the child to repeat completed trials. Participation was voluntary throughout, and children were not required to continue the task when they needed a rest.

### Data analysis

A 2 (test tool: Lego, geometric) × 3 (rotation angle: 0°, 90°, 180°) repeated-measures ANOVA was used to test the effects of test tool, rotation angle, and their interaction. Where Mauchly’s test of sphericity was significant, Greenhouse–Geisser corrections were applied; *post hoc* pairwise comparisons used Bonferroni corrections, and partial eta squared (ηp^2^) is reported as the effect size for the ANOVAs. To further examine the angle effect under each tool, separate one-way repeated-measures ANOVAs were conducted for the Lego and geometric conditions. Pearson correlation analysis was used to examine the association between the two tool totals.

The egocentric-error analysis included only the 90° and 180° conditions, and the proportion of egocentric errors was computed separately for Lego 90°, Lego 180°, geometric 90°, and geometric 180°. A 2 (test tool: Lego, geometric) × 2 (rotation angle: 90°, 180°) repeated-measures ANOVA tested the tool and angle effects and their interaction; where the interaction was significant, Bonferroni-corrected simple-effects analyses were conducted, and Cohen’s d is reported. In addition, a supplementary mixed-design ANOVA of 2 (tool) × 3 (angle) × 2 (sex) included sex as a between-subject variable.

## Results

### Interactive effects of test tool and rotation angle on VPT-2 performance

Descriptive statistics for the different tool and angle conditions are shown in Table 1, and the results of the 2 (test tool: Lego, geometric) × 3 (rotation angle: 0°, 90°, 180°) repeated-measures ANOVA in Table 2. Because the main effect of angle violated the sphericity assumption, its results were evaluated with the Greenhouse–Geisser correction.

**Table 1:** Descriptive statistics for VPT-2 scores by test tool and rotation angle (*N* = 163).

Condition	*M*	*SD*
Lego 0°	2.18	0.80
Lego 90°	1.34	0.93
Lego 180°	1.80	0.94
Geometric 0°	1.66	0.90
Geometric 90°	0.82	0.87
Geometric 180°	1.04	0.90

Each Tool × Angle condition comprised 3 formal trials, so condition scores ranged from 0 to 3. M and SD are the mean and standard deviation of raw correct scores, not percentage correct. All children included in the analyses completed all 18 formal trials.

**Table 2:** Summary of analysis-of-variance results.

Effect	*F*	*df*	*p*	ηp^2^	Pattern
2 × 3 analysis					
Tool (main effect)	125.279	(1, 162)	< 0.001	0.436	Lego > geometric
Angle (main effect, G–G corrected)	72.383	(1.920, 311.103)	< 0.001	0.309	0° > 180° > 90°
Tool × Angle	2.397	(2, 324)	0.093	0.015	ns
Separate analyses					
Lego: angle main effect	44.074	(2, 324)	< 0.001	0.214	0° > 180° > 90°
Geometric: angle main effect	38.747	(2, 324)	< 0.001	0.193	0° > 90°; 0° > 180°; 180° vs. 90° weak/marginal

G–G = Greenhouse–Geisser correction. The separate analyses were used to verify the robustness of the angle effect across tools.

The main effect of test tool was significant, *F*(1, 162) = 125.279, *p* < 0.001, ηp^2^ = 0.436, with higher scores in the Lego condition (*M* = 1.77, *SD* = 0.74) than in the geometric condition (*M* = 1.17, *SD* = 0.72). The main effect of rotation angle was significant, *F*(1.920, 311.103) = 72.383, *p* < 0.001, ηp^2^ = 0.309. Bonferroni-corrected pairwise comparisons showed that all three angle conditions differed significantly (all *ps* < 0.001), with 0° (*M* = 1.92, *SD* = 0.88) > 180° (*M* = 1.42, *SD* = 0.97) > 90° (*M* = 1.08, *SD* = 0.93). The Tool × Angle interaction did not reach significance, *F*(2, 324) = 2.397, *p* = 0.093, ηp^2^ = 0.015.

Analyses conducted separately by tool showed a significant main effect of angle in the Lego condition, *F*(2, 324) = 44.074, *p* < 0.001, ηp^2^ = 0.214, with 0° > 180° > 90° and all pairwise differences significant (all *ps* < 0.001). The main effect of angle was likewise significant in the geometric condition, *F*(2, 324) = 38.747, *p* < 0.001, ηp^2^ = 0.193, where 0° exceeded both 90° and 180° (both *ps* < 0.001), and the Bonferroni-corrected comparison between 90° and 180° yielded *p* = 0.050. The two tool totals were weakly positively correlated, *r* = 0.244, *p* = 0.002, *N* = 163.

### Tool- and angle-specific distribution of egocentric errors

To examine whether children tended to choose the option corresponding to their own viewpoint under incongruent-viewpoint conditions, this analysis excluded the trials with a 0° rotation angle—that is, it asked whether, when the participant and the observer held different viewpoints, the participant still chose the picture seen from their own position. Egocentric errors were operationally defined, in non-0° trials, as the selection of the photograph corresponding to the child’s own viewpoint (rather than the photographer’s). Specifically:

#### 90° condition

The photographer stood directly to the child’s left or right, in an orthogonal relation to the child’s viewpoint. For example, the child faced the front of the object and saw the frontal view, whereas the photographer saw a side view (left or right). Among the four options, the frontal photograph matching the child’s current viewpoint served as the egocentric distractor.

#### 180° condition

The photographer faced the child, with fully opposing viewpoints. For example, what the child saw as the front was the photographer’s back, and vice versa. Among the four options, the photograph matching the child’s current viewpoint served as the egocentric distractor.

Because the Lego and geometric tasks differ in task structure and stimulus features, the egocentric errors of the two tools were not simply pooled; instead, the proportions of egocentric errors were computed separately for Lego 90°, Lego 180°, geometric 90°, and geometric 180°. Each condition comprised 3 trials, so each child’s number of egocentric errors ranged from 0 to 3 per condition; across all non-0° trials, the theoretical range was 0 to 12.

As shown in Table 3, the geometric 180° condition produced the highest proportion of egocentric errors (50.10%), and 80.98% of children made at least one egocentric error; the error proportions were 31.70% in the Lego 180° condition, 28.83% in the Lego 90° condition, and 23.31% in the geometric 90° condition. A further 2 (test tool: Lego, geometric) × 2 (rotation angle: 90°, 180°) repeated-measures ANOVA (Table 4) showed a significant main effect of tool, *F*(1, 162) = 7.061, *p* = 0.009, ηp^2^ = 0.042; a significant main effect of angle, *F*(1, 162) = 52.501, *p* < 0.001, ηp^2^ = 0.245; and a significant Tool × Angle interaction, *F*(1, 162) = 34.794, *p* < 0.001, ηp^2^ = 0.177.

**Table 3:** Descriptive statistics for egocentric errors by test tool and rotation angle.

Condition	Trials	Total responses	Egocentric errors	Proportion	n with ≥1 error (%)	Individual *M* ± *SD* error proportion
Lego 90°	3	489	141	28.83%	88 (53.99%)	0.288 ± 0.318
Lego 180°	3	489	155	31.70%	108 (66.26%)	0.317 ± 0.284
Geometric 90°	3	489	114	23.31%	75 (46.01%)	0.233 ± 0.297
Geometric 180°	3	489	245	50.10%	132 (80.98%)	0.501 ± 0.330

Each condition comprised 3 trials, and *N* = 163, so each condition totaled 489 responses. The error proportion is the number of egocentric errors divided by the total responses in that condition; the individual error proportion is the mean and standard deviation, across children, of each child’s egocentric-error proportion in that condition.

**Table 4:** 2 × 2 repeated-measures ANOVA of the proportions of egocentric errors: test tool × rotation angle.

Source	*SS*	*df*	*MS*	*F*	*p*	ηp^2^
Tool	0.676	1	0.676	7.061	0.009	0.042
Error (tool × participant)	15.518	162	0.096			
Angle	3.583	1	3.583	52.501	< 0.001	0.245
Error (angle × participant)	11.056	162	0.068			
Tool × angle	2.333	1	2.333	34.794	< 0.001	0.177
Error (tool × angle × participant)	10.862	162	0.067			

Simple-effects analyses used the Bonferroni correction, with a corrected significance level of *α* = 0.0125; ηp^2^ is partial eta squared.

Given the significant Tool × Angle interaction, Bonferroni-corrected simple-effects analyses were conducted. The results showed that, in the geometric condition, the proportion of egocentric errors was significantly higher at 180° than at 90°, *t*(162) = −8.435, *p* < 0.001, d = −0.661; and that, at 180°, the geometric condition produced a significantly higher proportion of egocentric errors than the Lego condition, *t*(162) = −5.649, *p* < 0.001, d = −0.442. Neither the difference between 90° and 180° in the Lego condition nor the difference between the two materials at 90° reached the Bonferroni-corrected significance level.

### Supplementary analysis: sex

The supplementary analysis found no significant main effect of sex, *F*(1, 161) = 0.674, *p* = 0.413, ηp^2^ = 0.004. The Tool × Sex interaction, *F*(1, 161) = 2.042, *p* = 0.155, ηp^2^ = 0.013; the Angle × Sex interaction, *F*(1.922, 309.440) = 2.231, *p* = 0.111, ηp^2^ = 0.014; and the Tool × Angle × Sex interaction, *F*(1.958, 315.297) = 0.493, *p* = 0.607, ηp^2^ = 0.003, likewise failed to reach significance. No further sex-related *post hoc* comparisons were therefore conducted.

## Discussion

### The effect of rotation angle on task performance

The repeated-measures ANOVA showed a significant main effect of rotation angle, with significant differences among all three angle conditions. Specifically, the 0° baseline (no perspective transformation) yielded the highest scores, followed by the 180° condition, with the 90° condition lowest. This result indicates that, in the present task, 4-year-olds’ VPT-2 performance did not simply follow the linear pattern of “the larger the rotation angle, the harder the task,” but instead showed a nonmonotonic angle effect of 0° > 180° > 90°.

Notably, the Tool × Angle interaction did not reach significance (*p* = 0.093), indicating that this study observed no significant moderation of the angle effect by test tool. In other words, although the Lego blocks and geometric solids clearly differed in overall difficulty, the direction of the angle effect was broadly consistent under the two tools. However, because a nonsignificant interaction cannot prove that the angle effects are identical under the two tools, and because sample size or statistical power may have been limiting, this result should be interpreted with caution: the nonmonotonic angle effect showed a similar trend under both material conditions, rather than being an isolated phenomenon specific to one material.

Surtees et al. (2013) proposed that Level 2 visual or spatial perspective-taking tasks typically involve a transformation from one’s own viewpoint to another’s. The “picture-taking game” used in this study is a Level 2 visual perspective-taking task and is thus theoretically related to this transformation process. The present results further show that, in a sample of 4-year-olds, task performance differed markedly across rotation angles, with 90° significantly lower than 180°. This finding suggests that young children’s performance on Level 2 tasks may depend not only on whether perspective transformation is required but also on the specific viewpoint relation; angle condition may thus constitute an important task variable in young children’s VPT-2.

It must be emphasized that this study collected only behavioral raw-correct-score data and did not directly measure children’s processing strategies, response times, eye movements, or bodily simulation processes. The present results therefore cannot directly show that children actually adopted different strategies at 90° and 180°, nor whether they performed mental self-rotation. The more conservative interpretation is that the lower performance at 90° is consistent with the explanation that this angle may impose higher spatial-transformation demands on young children—an interpretation that awaits further testing in future research.

Several possible explanations can be offered for why the 90° condition was more difficult than the 180° condition. First, at 90° the self and other perspectives are orthogonally related, and children may find it harder to transform their own frame of reference into the photographer’s (Muto et al., 2019). Second, the 180° relation is closer to everyday face-to-face interaction, and children may find it easier to draw on existing experience; by comparison, the lateral 90° viewpoint may lack equally stable experiential support in daily interaction (Vander Heyden et al., 2017). Third, prior research suggests that older children may use different strategies at different angles, so 4-year-olds’ difficulty at 90° may also relate to immature strategy selection (Vander Heyden et al., 2017). These explanations, however, are *post hoc* conjectures based on behavioral performance and are not mechanisms directly tested in this study.

### The test-tool effect and its cross-angle consistency

The results showed a significant main effect of test tool with a large effect size (ηp^2^ = 0.436): performance was significantly better in the Lego figurine condition than in the geometric solids condition. However, because the two conditions differ simultaneously on three dimensions—task structure (single-object orientation judgment vs. two-object spatial-relation judgment), stimulus discriminability (highly discriminable figurines vs. geometric solids containing rotationally and contour-symmetric objects), and social cues—this study cannot attribute the tool effect purely to social cues. The following discussion explores possible explanatory directions while acknowledging this methodological limitation.

First, the task-structure hypothesis. The Lego condition required participants to judge the orientation of a single figurine (front/back/left side/right side), whereas the geometric condition required them to judge the spatial layout of two objects (e.g., their left–right relation); the latter involves reasoning about and memory for the spatial relations of multiple objects and thus imposes a markedly higher cognitive load. Frick et al. (2014) found that when the number of objects increased, 4- and 5-year-olds’ performance declined significantly on trials requiring perspective transformation. Although the geometric condition in this study presented only two objects, it already constituted a spatial-relation judgment task more difficult than single-object orientation judgment. The Lego advantage may therefore mainly reflect a reduction in cognitive load arising from task-structure differences.

Second, the stimulus-discriminability hypothesis. In the geometric condition, the side silhouette of the cylinder is nearly invariant under rotation about the vertical axis (always a rectangle); the side silhouette of the cone is highly similar across horizontal viewpoints (always a triangle); and all faces of the cube are squares, yielding identical outlines under orthographic projection. Only the rectangular prisms, with their aspect-ratio differences, offer clearly discriminable viewpoints. Some trials additionally paired same-colored objects (e.g., a yellow cone with a yellow cylinder), further reducing visual discriminability. By contrast, the Lego figurine’s face, limbs, and clothing details differ markedly across viewpoints.

Third, the social-cue hypothesis (to be tested). After controlling the confounds above, social cues (face, human form, orientation) might still independently facilitate VPT-2 performance, perhaps through the activation of social-cognitive modules or the facilitation of embodied simulation (Gunalp et al., 2019). The present design, however, cannot isolate this effect. Future research should adopt the following comparison designs: (a) task-structure matching—a single geometric object with an explicit front and back (e.g., an asymmetric arrow) versus a single figurine, holding task structure constant; (b) social-cue separation—objects retaining the human-like contour but without a face versus intact figurines, isolating the face effect; and (c) discriminability control—a single object with a clear orientation but no social cues (e.g., a geometric body with directional markers), testing the independent role of orientation cues. Only after these confounds are controlled can firm conclusions be drawn about the role of social cues.

#### Interpreting the tool × angle interaction

The Tool × Angle interaction was not significant (*p* = 0.093), indicating broadly consistent patterns of the angle effect across the two conditions. Given that the tool effect itself is multiply confounded, the interpretation of this interaction is likewise limited. If future research, after controlling task structure and stimulus discriminability, still finds the interaction nonsignificant, the conclusion that social cues facilitate performance consistently across angles would receive stronger support.

#### Methodological implications

The Lego and geometric test scores were weakly correlated (*r* = 0.244, *p* = 0.002, r^2^ = 0.06), indicating that the two tools share only about 6% of their variance. Combined with the significant tool main effect and the confound analysis above, this pattern suggests that the two conditions may measure different cognitive processes (single-object perspective transformation vs. multi-object spatial-relational reasoning) rather than method variance of a single construct. Researchers comparing results across studies must therefore seriously consider the systematic bias introduced by task structure and stimulus features.

### Context dependence of egocentric errors

This study found that egocentric errors did not occur at similar proportions across all non-0° conditions, but instead showed clear tool and angle dependence, being most pronounced in the geometric 180° condition; in the Lego task, the difference in egocentric errors between 90° and 180° was not significant. This indicates that egocentric errors cannot simply be understood as a stable tendency common to children of a given age, nor be explained by rotation angle alone; they are more plausibly shaped jointly by task material, spatial-relational judgment demands, and viewpoint condition.

That the geometric 180° condition more readily elicited egocentric errors may be related to the structure of that task. Compared with the orientation judgment of a single figurine in the Lego task, the geometric task required children to judge the relative spatial relations of two objects. In the 180° condition, children had not only to re-encode the scene from the photographer’s viewpoint but also to handle the reversal of the left–right relation between the objects, which may have increased task load and made children more likely to choose the option corresponding to their own viewpoint. This study did not, however, measure response times, eye movements, working memory, or inhibitory control, and therefore cannot directly determine whether these errors stem from insufficient inhibitory control, difficulty in frame-of-reference transformation, or other processing mechanisms.

The conclusions of this study regarding egocentric errors should therefore be restricted as follows: 4-year-olds’ egocentric errors in VPT-2 tasks show clear task-context dependence, being most pronounced when geometric materials are combined with the 180° viewpoint. This result is not used to argue that “egocentrism lies on a continuum rather than being an all-or-none stage,” but rather shows that different task conditions significantly affect whether children choose the option corresponding to their own viewpoint. Future research seeking to examine the structure of individual differences in egocentrism will need more appropriate distributional tests—such as bimodality tests or latent class analysis—combined with more trials and independent measures of inhibitory control.

### Limitations and future directions

This study has the following limitations, which should be considered when interpreting the results.

First, the rotation angles were still limited. This study included only three angle conditions: 0°, 90°, and 180°. The strength of this design is that these angles represent three typical situations—no perspective transformation, orthogonal lateral transformation, and face-to-face reversal—and, in real experiments with young children, are easier to implement in terms of arranging physical materials, standardizing photographer positions, and explaining the instructions. For 4-year-olds, additional angle conditions would substantially increase the number of trials and the length of the session, potentially introducing fatigue, declining attention, or rule confusion as sources of extra error. The study therefore chose the three most representative angle conditions as a compromise between experimental feasibility and age-appropriateness. This design nonetheless limits the scope of interpretation of the angle effect. Because 45°, 135°, or other intermediate angles were not included, this study cannot test whether VPT-2 performance varies as a continuous function of angle, nor whether task difficulty changes gradually between 90° and 180°. The conclusion that “the 90° condition was lower than the 180° condition” should therefore be restricted to behavioral differences among three discrete angle conditions and should not be interpreted as direct evidence of a complete angular curve or of differences in processing strategies. Future research could add intermediate angles such as 45° and 135° while preserving task feasibility for young children, and could combine response times, eye tracking, body-posture manipulations, or strategy reports to examine more finely the relation between angular variation and VPT-2 processing.

Second, the statistical power for the Tool × Angle interaction. Although the main effects of tool and angle both reached significance, the Tool × Angle interaction fell short of conventional significance (*p* = 0.093, ηp^2^ = 0.015). A *post hoc* power analysis (G*Power 3.1) showed that, with the observed effect size (f* = 0.123) and the current sample size (*N* = 163), the achieved power was approximately 0.52; detecting this effect at 80% power would require about 350 participants. The present results therefore cannot rule out the possibility of a weak angle-specific facilitation by social cues, a question that awaits larger samples.

Third, the current analyses show only that egocentric errors display context dependence across tool and angle conditions, concentrating in the geometric 180° condition. If future research wishes to test whether egocentrism is continuously distributed, categorically differentiated, or bimodally structured, it should increase the number of trials, use strictly matched tool conditions, and apply direct tests such as bimodality tests, latent class analysis, or mixture models.

Fourth, confounds in the comparison of test tools. In this study, the Lego condition (single-object orientation judgment) and the geometric condition (two-object spatial-relation judgment) differ fundamentally in task structure; moreover, the geometric condition includes rotationally symmetric cylinders and cones, contour-symmetric cubes, and some trials pairing same-colored objects, so its stimulus discriminability is markedly lower than that of the Lego condition. Interpretation of the tool effect is therefore highly uncertain, and “task-structure differences” or “stimulus-discriminability differences” cannot be ruled out as alternative explanations. Future research should adopt the following improved designs: (a) task-structure matching—a single asymmetric geometric body (e.g., an arrow with a clear front and back) versus a single figurine, controlling task structure and set size; (b) social-cue separation—faceless human-like figures versus figurines with faces, isolating the face effect; and (c) systematic manipulation of discriminability—contrasting the same objects under different color and texture conditions. Only after these confounds are controlled can firm conclusions be drawn about the independent role of social cues.

Fifth, it should be noted that this study collected only behavioral raw-correct-score data and did not measure response times, eye movements, dual-task interference, or strategy reports. The finding that the 90° condition was lower than the 180° condition therefore indicates only differential difficulty across the two angle conditions and cannot directly show that children adopted different processing strategies. Future research combining process measures could further test whether processing strategies differ across angle conditions.

Sixth, the sample source and cross-cultural interpretation remain limited. The sample of this study comprised 4-year-olds from Hainan, China, with no Western comparison group, so no statistically direct comparison of VPT-2 performance between cultural groups is possible. The present results can serve only as a preliminary reference for future cross-cultural research, not as conclusive evidence of cross-cultural differences or differences in developmental level. To test the developmental patterns of VPT-2 tasks across cultural contexts, future research should use strictly matched paradigms, stimulus materials, task structures, and scoring criteria, with concurrent data collection in multiple cultural samples.

Seventh, future research could further combine theory of mind, inhibitory control, or process measures to examine the relations between VPT-2 performance and other social-cognitive and executive processes.

Eighth, this study did not systematically collect information on children’s family socioeconomic status (SES), such as parental education, occupation, and family income. The existing data therefore cannot test the relation between socioeconomic background and VPT task performance, nor rule out the potential influence of family-background differences on the results. Although this study used a within-subjects design, comparing each child’s performance across experimental conditions, it remains impossible to determine whether socioeconomic background moderates the effects of rotation angle or test material on task performance. Future research should include family SES indicators to examine task performance and condition effects among children from different backgrounds. In addition, children’s bilingual backgrounds and home language use were not systematically collected, so whether language background relates to task performance or to performance differences across experimental conditions remains a question for future research.

## Conclusion and theoretical implications

### Conclusion

Using a picture-taking task, this study examined the performance of 4-year-old children in Hainan, China, on Level-2 visual perspective-taking tasks, focusing on the effects of rotation angle and test tool and on the pattern of egocentric errors. Based on the results, the following conclusions can be drawn.

First, rotation angle significantly affected 4-year-olds’ VPT-2 task performance. Correct scores in the 0° condition were significantly higher than in the 90° and 180° conditions, consistent with the prediction of H1. Further exploratory comparisons found that correct scores in the 180° condition were significantly higher than in the 90° condition, yielding an overall nonmonotonic pattern of 0° > 180° > 90°. This indicates that, across the three angle conditions set in this study, children’s task performance did not decline monotonically as the rotation angle increased. It should be noted that this study analyzed raw correct scores only; the above results should therefore be understood as behavioral differences across angle conditions and cannot directly demonstrate that children adopted different cognitive processing strategies.

Second, the test tool significantly affected task performance, and the effect was consistent across angles, in line with the directional prediction of H2. However, because the Lego and geometric conditions are confounded on three dimensions—task structure (single-object orientation judgment vs. two-object spatial-relation judgment), stimulus discriminability (geometric solids with rotational symmetry, contour symmetry, and same-color objects vs. highly discriminable figurines), and social cues—the Lego advantage may reflect the joint contribution of task-structure differences, stimulus discriminability, and social cues. The weak positive correlation between the Lego and geometric test scores (*r* = 0.244) may partly reflect that the two conditions measure different cognitive processes rather than method variance of a single construct. Future research will need designs with matched task structure and controlled stimulus discriminability to separate these factors.

Third, in the raw-correct-score analysis, the Tool × Angle interaction did not reach significance. This indicates that, although overall performance was better in the Lego task than in the geometric task, this study observed no evidence that the tool effect varied significantly with rotation angle. In other words, the overall trend of the Lego task outperforming the geometric task appeared at 0°, 90°, and 180° alike, but the variation of this difference across angles did not reach statistical significance. This study therefore does not treat the Tool × Angle interaction as a supported directional hypothesis, but reports it only as an exploratory test within the model.

Fourth, egocentric errors showed clear tool and angle dependence. Tool-specific analyses showed that egocentric errors did not occur at similar proportions across all non-0° conditions, but were most pronounced in the geometric 180° condition. This result indicates that children’s tendency to choose the option corresponding to their own viewpoint is related not only to rotation angle but also jointly to test tool, task structure, and spatial-relational judgment demands. This study no longer interprets this result as an “egocentrism continuum” or as a direct revision of stage theory, but restricts it to an error pattern under specific task contexts.

The supplementary sex analysis found no significant sex main effect or sex-related interactions. Because sex was not a core research question of this study, no further interpretation is offered of the descriptive differences between boys and girls in specific angle conditions, nor are the sex results treated as primary conclusions.

Overall, this study provides behavioral evidence for the angle specificity and task-context dependence of 4-year-olds’ VPT-2 task performance. The findings suggest that, when 4-year-olds understand others’ viewpoints, performance is influenced not only by viewing angle but also by task material, stimulus discriminability, and spatial-relational judgment demands. Future research should use more strictly matched material designs and combine process measures—response times, eye tracking, strategy reports, and inhibitory-control measures—to further examine the cognitive mechanisms underlying different angles and different task materials.

### Theoretical implications

The theoretical implications of this study are reflected mainly in three aspects.

First, this study provides behavioral evidence for angle effects in 4-year-olds’ VPT-2 tasks. Prior research generally holds that Level-2 visual perspective taking depends more on perspective transformation than Level-1 visual perspective taking, but whether different rotation angles impose the same difficulty has required further examination. This study found that 4-year-olds performed differently at 0°, 90°, and 180°, with the 90° condition lower than the 180° condition, indicating that the difficulty of VPT-2 tasks does not simply increase linearly with rotation angle. This result suggests that, in research on young children’s Level-2 visual perspective taking, it is necessary to distinguish angle conditions rather than to merge all non-0° conditions into a single category of “transformation-required” tasks.

It must be emphasized that this study did not directly measure mental rotation, egocentric transformation, bodily simulation, or strategy use. The current results therefore cannot show that children adopted different processing strategies at 90° and 180°, nor can they directly speak to the neural or embodied mechanisms of the angle effect. The more cautious interpretation is that 4-year-olds show different degrees of behavioral difficulty across angle conditions, with the underlying processing mechanisms awaiting further examination in future research through process measures such as response times, eye tracking, body-posture manipulations, or strategy reports.

Second, this study provides complementary evidence for the findings of Frick et al. (2014) on task complexity and angle effects. Frick et al. (2014) found an interaction between spatial complexity and rotation angle, with the complexity effect concentrated in the 90° and 180° trials requiring perspective transformation, and uniformly low performance by 4-year-olds on perspective-transformation trials. The present study likewise focused on 4-year-olds and found that the test tool significantly affected task performance, with children performing better in the Lego figurine condition than in the geometric solids condition.

The “tool effect” in this study, however, cannot be equated with the “spatial complexity effect” of Frick et al. The Lego and geometric tasks differ not only in material appearance but also simultaneously in task structure, stimulus discriminability, and social cues. This study is therefore better understood as showing that VPT-2 performance is task-context dependent, rather than as demonstrating an independent facilitative role of any single material factor or of social cues. At the same time, the correct-score analysis and the error-type analysis yielded different pictures: in raw correct scores, tool differences appeared mainly as an overall tool effect, whereas in egocentric errors, tool and angle showed clearer condition dependence. This suggests that future research on the influence of task materials should attend not only to accuracy but also to error types.

Third, this study conducted a more fine-grained, conditionalized analysis of egocentric errors. Traditional accounts often interpret preschool children’s egocentric errors as a manifestation of preoperational children’s difficulty in leaving their own viewpoint. The present results do not directly contradict this classic view, but they suggest that the strength with which egocentric errors emerge may be influenced by specific task conditions. The highest proportion of egocentric errors in the geometric 180° condition indicates that whether children choose the option corresponding to their own viewpoint may depend jointly on task material, spatial-relational judgment demands, and viewpoint condition.

This study should therefore not be framed as a direct revision or alternative interpretation of Piaget’s stage theory, but is better treated as complementary behavioral evidence: 4-year-olds’ egocentric errors do not occur uniformly across VPT-2 conditions but show clear sensitivity to task conditions. Future research, building on more trials, more strictly matched materials, and richer individual-difference measures, could further examine the cognitive origins of egocentric errors and their developmental changes.

## Data Availability

The original contributions presented in the study are included in the article/supplementary material, further inquiries can be directed to the corresponding authors.
